# The Potential of Chitosan in Nanomedicine: An Overview of the Cytotoxicity of Chitosan Based Nanoparticles

**DOI:** 10.3389/fphar.2022.880377

**Published:** 2022-05-04

**Authors:** Julie Frigaard, Janicke Liaaen Jensen, Hilde Kanli Galtung, Marianne Hiorth

**Affiliations:** ^1^ Department of Oral Surgery and Oral Medicine, Institute of Clinical Odontology, University of Oslo, Oslo, Norway; ^2^ Institute of Oral Biology, University of Oslo, Oslo, Norway; ^3^ Section for Pharmaceutics and Social Pharmacy, Department of Pharmacy, The Faculty of Mathematics and Natural Sciences, University of Oslo, Oslo, Norway

**Keywords:** chitosan, nanoparticles, drug carriers, drug delivery systems, cytotoxicity, cell viability, nanocapsule

## Abstract

The unique properties and applications of nanotechnology in targeting drug delivery, cosmetics, fabrics, water treatment and food packaging have received increased focus the last two decades. The application of nanoparticles in medicine is rapidly evolving, requiring careful investigation of toxicity before clinical use. Chitosan, a derivative of the natural polysaccharide chitin, has become increasingly relevant in modern medicine because of its unique properties as a nanoparticle. Chitosan is already widely used as a food additive and in food packaging, bandages and wound dressings. Thus, with an increasing application worldwide, cytotoxicity assessment of nanoparticles prepared from chitosan is of great interest. The purpose of this review is to provide an updated status of cytotoxicity studies scrutinizing the safety of chitosan nanoparticles used in biomedical research. A search in Ovid Medline from 23 March 1998 to 4 January 2022, with the combination of the search words *Chitosan* or *chitosan*, *nanoparticle* or *nano particle* or *nanosphere* or *nanocapsule* or *nano capsule*, *toxicology* or *toxic* or *cytotoxic* and *mucosa* or *mucous membrane* resulted in a total of 88 articles. After reviewing all the articles, those involving non-organic nanoparticles and cytotoxicity assays conducted exclusively on nanoparticles with anti-tumor effect (i.e., having cytotoxic effect) were excluded, resulting in 70 articles. Overall, the chitosan nanoparticles included in this review seem to express low cytotoxicity regardless of particle composition or cytotoxicity assay and cell line used for testing. Nonetheless, all new chitosan derivatives and compositions are recommended to undergo careful characterization and cytotoxicity assessment before being implemented on the market.

## 1 Nanotechnology and Chitosan

Nanotechnology is rapidly expanding, and the global nanotechnology market has increased its market value by tenfold, from 1.8 billion USD in 2020 to an expected level of more than 33 billion USD in 2030 (Divyanshi Tewari, 2019). Properties of nanomaterials may differ from bulk material, because of their small size, large surface area and polydispersity. Compared to bulk particles with <1% of total atoms on the surface, >80% of total atoms are on the surface of nanoparticles (NPs), offering new biological properties (Singh, 2016). Thus, the surface atoms will influence particle properties and size, and lead to shape-dependent physicochemical properties (Singh, 2016).

Chitosan, a derivative of the natural polysaccharide chitin, is the second most abundant polysaccharide in the world, after cellulose. Because of properties like biocompatibility, biodegradability, antibacterial effect and muco-adhesion, chitosan is widely used in food, cosmetics, fabrics, water treatment and biomedical applications (Elieh-Ali-Komi and Hamblin, 2016). The United States Food and Drug Administration (US-FDA) and EU have approved chitosan as a food additive, fat absorption material and wound dressing (Mohammed et al., 2017). Chitosan and its derivatives are found in several products on the market today, such as food additives (LipoSan Ultra^TM^, Primex), cosmetics (ChitoCare^TM^, Primex), antibacterial agents (Chitocell^TM^, ChitoTech), haemostatic dressings (Axiostat^TM^, Axio), wound healing materials (Opticell^TM^, Medline) and oral solutions (Moisyn^TM^, Prisyna). The global chitosan market is estimated to have an annually growth of 25% between 2020 and 2027, which will result in a market size of 29 billion USD in 2027 (Grand View Research, 2020).

Previous *in vivo* toxicity studies on chitosan as bulk material show low toxicity, but nanoparticles possess new biological properties such as high surface-to-area ratio, thus new safety evaluations are called for. The purpose of this review is to provide an updated status on the toxicity of chitosan nanoparticles used in biomedical research.

A search in Ovid Medline, a search engine specialized for biomedical research, at 4 January 2022 with the search words *chitosan* showed 24,793 results, after specifying the search by combining the words *Chitosan* or *chitosan*, *nanoparticle* or *nano particle* or *nanosphere* or *nanocapsule* or *nano capsule*, *toxicology* or *toxic* or *cytotoxic* and *mucosa* or *mucous membrane,* the result was 88 articles. After applying the exclusion criteria *non-organic nanoparticles* and studies that evaluated cytotoxicity only of nanoparticles incorporated with anti-tumor effect (i.e., having cytotoxic effect), a set of 70 articles remained to be included and discussed in this overview.

## 2 Chitin, Chitosan and Chitosan Nanoparticles

**FIGURE 1:**
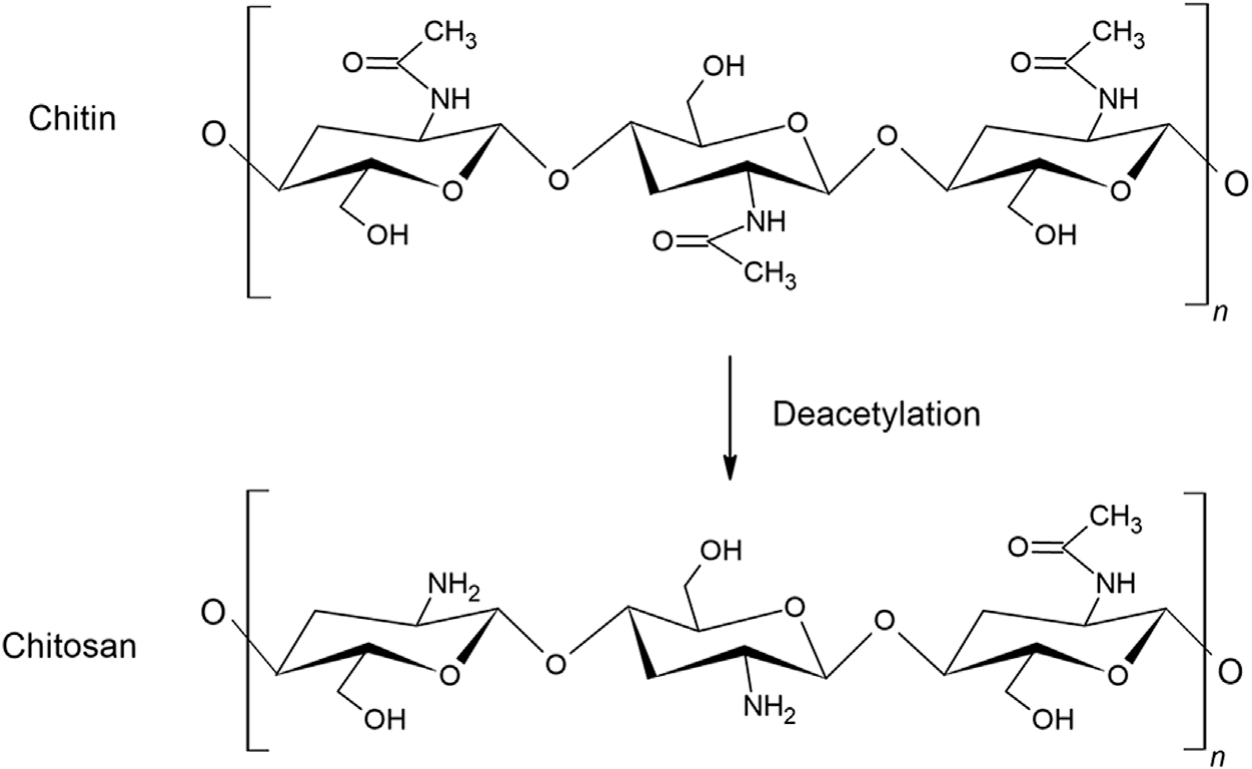
The conversion of chitin to chitosan by N-deacetylation.

Chitin is a natural polysaccharide consisting of the two monosaccharides N-acetyl-D-glucosamine and D-glucosamine, connected by β-1,4-glycosidic bonds. Chitin is mainly found in oceans as a constituent of shells and crustaceans, but is also found in insects, algae, bacteria and fungi. Chitin has a supporting function in cell walls and exhibits many of the same functions as cellulose. The most common sources of commercial chitin are crab and shrimp shells, and it can therefore conveniently be prepared from wastes of seafood processing industries. The content of chitin ranges from 6 to 72% in crustacean shells, crabs and shrimps, dependent on the species (van den Broek et al., 2020). The isolation of chitin is relatively time and energy consuming and is environmentally polluting as it involves hazardous chemicals. The shell isolation process may vary depending on species, but consists mainly of washing, drying, demineralization with hydrochloric acid (HCl) and deproteination with sodium hydroxide before removing pigments (Kurita, 2001). Chitin is insoluble in many solvents, and great attention has been given to convert chitin into more soluble derivatives, the simplest modification being N-deacetylation, which converts chitin into chitosan (Figure 1).

Chitosan has the ability to interact electrostatically with negatively charged molecules, such as cells, nanoparticles, lipids, drugs and polymers because of the functional amino groups on the surface of the molecule (Nurunnabi et al., 2017). The pKa of chitosan is 6.3 and consequently it is soluble in acidic solutions and insoluble in basic conditions, and at pH 6.0–6.5 chitosan will self-aggregate (Kumar et al., 2004). Since only the non-acetylated amino groups are able to bind protons, the solubility of chitosan is mainly dependent on the degree of deacetylation (number of glucosamine units after deacetylation), but also on the ionic strength and the distribution of acetyl groups along the chain (Berth and Dautzenberg, 2002). The reactivity of chitosan is mainly affected by the molecular weight, degree of deacetylation and pH (Jana and Jana, 2020).

Nanoparticles are particles of small size, from 1 to 100 nanometers (nm), but the term is often used for larger particle sizes described in nm. Active substances encapsulated in nanoparticles are concealed from its surroundings, and can be transported incognito to specific sites, depending on the nanoparticle surface properties. Chitosan nanoparticles are especially interesting because of their mucoadhesive properties, positive surface charge and ability to open tight junctions between cells (Liu et al., 2008; Nurunnabi et al., 2017). In medical research, chitosan nanoparticles are promising agents as targeted delivery vehicles for drugs, adjuvants and delivery carriers for vaccines (Prabaharan and Mano, 2005; Amidi et al., 2010). Chitosan nanoparticles are of great interest as oral drug carriers for proteins, as they are capable of preventing enzymatic degradation in the gastrointestinal system and facilitating mucoadhesion to the intestinal mucus layer (Janes et al., 2001; Amidi et al., 2010). Several articles in this review investigated the use of chitosan nanoparticles in ocular-targeted drug delivery, drug delivery over the blood-brain barrier, targeted delivery of bio-imaging markers and vaccination by oral- and intranasal administration (de Campos et al., 2004; Amidi et al., 2006; Borges et al., 2006; Diebold et al., 2007; Sayin et al., 2008; Saremi et al., 2011; Cheng et al., 2012; Patel et al., 2012; Chen et al., 2013a; Dehghan et al., 2013; Ye et al., 2013; Zhao et al., 2014; Liu et al., 2015; da Silva et al., 2016; Shah et al., 2016; Bor et al., 2017; Jin et al., 2017; Pandit et al., 2017; Shi et al., 2017; Zhao et al., 2017; Çelik Tekeli et al., 2018; Cole et al., 2018; Tandberg et al., 2018; Bento et al., 2019; Sinani et al., 2019; Tzeyung et al., 2019). A considerable amount of research on chitosan nanoparticles in cancer medicine has also been conducted, in order to decrease the side effects by encapsulating chemotherapeutics in chitosan nanoparticles, and to enhance the oral bioavailability of anti-cancer drugs (Akhlaghi et al., 2010; Guo et al., 2013; Battogtokh and Ko, 2014; Jain et al., 2015; Khan et al., 2019). Chitosan can act as coating material together with other materials or be the core material in the nanoparticle itself (nanosphere or nanocapsule).

## 3 Cytotoxicity Measurements

Cytotoxicity studies are divided into *in vitro-* and *in vivo* studies, depending on whether the study is performed on cultured cells or tissues in the laboratory or in live animals, respectively. Some of the factors that influence the choice of cytotoxicity methods are exposure duration, amount and frequency of substance exposure, the type of exposed tissues and results from previous toxicity studies. It is generally accepted that animal testing should be replaced with *in vitro* studies as far as possible for ethical considerations, but it may still be necessary to evaluate animal testing in specific end-points. The most used *in vitro* cytotoxicity methods in the included studies are different assays based on colorimetric readings of cell activity, with the MTT-assay (3-(4,5-dimethylthiazol-2-yl)-2,5-diphenyltetrazolium bromide reagent assay) being the far most frequently used. For the *in vivo* studies, clinical investigation such as weight, appetite and behavior, in addition to macroscopic and histologic assessment of the test animals, are the most frequently used methods.

## 4 Results of Cytotoxicity Measurements on Chitosan Containing Nanoparticles

**FIGURE 2:**
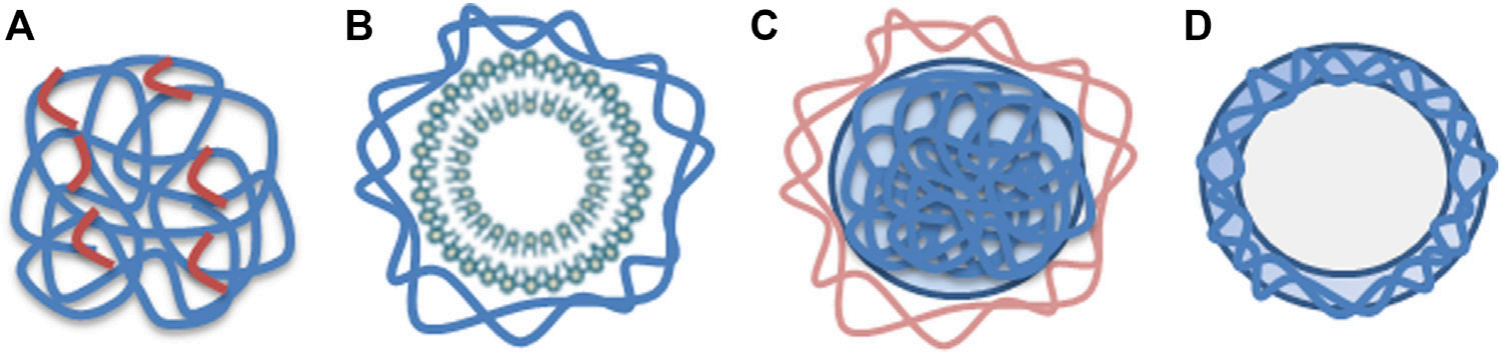
**(A)** Nanosphere composed of chitosan (blue) with crosslinkers (red), **(B)** Liposome (green) with chitosan coating (blue), **(C)** Chitosan nanoparticle (blue) covered with other substance (light brown) such as proteins or polymers, **(D)** Nanocapsule made of chitosan (blue).

Cytotoxicity studies regarding nanoparticles containing chitosan are presented below. The nanoparticle composition and chitosan type vary significantly in the selection of articles. Four different nanoparticle structures frequently mentioned in the articles are illustrated in Figure 2. In the following presentation, the articles are categorized into sections according to the nanoparticle composition. Each chapter includes a summary of the main findings concerning cytotoxicity of the specific group of nanoparticles, and a table of the main features from articles included in the section. The tables are sorted by molecular weight (MW), from small to large, and by study design (*in vitro*/*in vivo*). The first two sections present *chitosan as a nanoparticle*, with and without tripolyphosphate (TPP) as crosslinker, which constitute the major group in this review. The two next sections present *chitosan in combination with liposomes* and *nanoparticles coated with chitosan*. The following three sections include three of the most common derivatives of chitosan; *carboxymethylated-, quaternizied- and thiolated chitosan*. The last section describes *other derivatives and complexes* of chitosan nanoparticles.

### 4.1 Chitosan Nanoparticles With Tripolyphosphate as Crosslinker

For cytotoxicity of chitosan nanoparticles with TPP as crosslinker 25 articles were retrieved, one *in vivo*-, four *ex vivo*- and 20 *in vitro* studies. The main findings from the articles concerning the chitosan nanoparticles with TPP as crosslinker are presented in Table 1. Four of the articles investigated the cytotoxicity of chitosan nanoparticles using Caco-2 cells (human colorectal adenocarcinoma cells) and the MTT-assay. All studies showed good cell viability (>80%) for particles ranging from 126 to 1,000 nm (Zheng et al., 2011; Loh et al., 2012; Jain et al., 2015; Je et al., 2017). In one of the studies, the cell viability was lower in pH 6 than in pH 7.4. The surface charge was approximately the same, but the particle size was significantly smaller in pH 6 (25 ± 7 nm, 5.3 ± 2.8 mV) than in pH 7.4 (333 ± 43 nm, 3.3 ± 0.4 mV) (Loh et al., 2012). The authors suggested that particle size had more influence on the cytotoxicity in Caco-2 cells than the positive surface charge, because of easier cellular uptake of small particles than larger ones (Loh et al., 2012). This is in accordance with Zheng et al. (2011) who showed that chitosan nanoparticles as compared to chitosan molecules accumulated to a higher extent intracellularly, but in spite of high intracellular concentration of chitosan nanoparticles, the Caco-2 cells showed good viability. Another study reported no difference in cytotoxicity when comparing chitosan nanoparticles of increasing size from 200 to 1,000 nm (Je et al., 2017). These results may indicate that size-dependent cytotoxicity may be more profound when considering nanoparticles in the lower range (<200 nm) (Rejman et al., 2004). But the suggestion may be reserved for Caco-2 cells, as another study of Loh et al. (2010) showed >90% cell viability for human liver cells (BHAL) after incubation with chitosan nanoparticles of 18 and 25 nm.

**TABLE 1:** Articles on chitosan nanoparticles with TPP as crosslinker, main findings.

Chitosan characteristics	Nanoparticle composition	Active ingredient	Cell line/Species	Toxicity assay	Cytotoxicity results	References
Chitosan 80 kDa DDA ∼85%	Chitosan/TPP/MgSO_4_/poly-ɣ-glutamic acid	Insulin	Adult ICR mice	Clinical hematological biochemical histology	The unloaded NPs were well tolerated.	Sonaje et al. (2009)
Chitosan low MW DDA 85%	Chitosan/TPP	Hydrochlorothiazide (HCT)	Intestinal gut sac from wistar male rats	Histology	HCT-loaded NPs showed less prominent changes than free HCT.	Onnainty et al. (2016)
Chitosan ∼50 kDa DDA 86%	Chitosan/TPP	Rosmarinic acid, salvia officinalis (sage) and satureja montana (savory)	ARPE-19	MTT assay LDH assay HET-CAM	Rosmarinic acid-, Saliva officinalis- and Satureja montana loaded NPs: LDH assay: LDH assay: <10% cytotoxicity, MTT-assay: Non-toxic for concentrations <1 mg/ml, HET-CAM: Non-irritating.	da Silva et al. (2016)
Chitosan low MW DDA ≥75%	Chitosan/TPP, coated with retrograded soluble starch or retrograded high amylose corn starch	Doxorubicin and neutraceutical-coagulants	Caco-2 cells	CCK-8 kit	Unloaded NPs had no effect on cell viability after 2 h, 10%–15% cell death after 24 h.	Sampathkumar and Loo, (2018)
Chitosan DDA 95%	Chitosan/TPP/carrageenan	Resveratrol, coumarin-6	Caco-2 cells	MTT assay	Resveratrol-loaded NPs showed >90% cell viability for all sizes (200–1,000 nm).	Je et al. (2017)
Chitosan low MW	Chitosan/TPP	Inactivated influenza virus	Calu-6 cells	XTT assay	Unloaded dry-powder chitosan nanoparticles of 50, 250, and 500 µg/ml showed concentration dependent cell viability, from 100 to 70% after 2 h, and 60%–20% after 24 h.	Dehghan et al. (2013)
Chitosan Hydrochloride DDA 91.1%	Chitosan/TPP	Thymopentin	Caco-2 cells	MTT assay	Unloaded NPs showed 80%–90% viability in all tested concentrations (0.25, 0.5, 1.0, 1.5 and, 2.0 mg/ml) after 4 h.	Zheng et al. (2011)
Chitosan oligosaccharide ∼3 kDa DDA 90%	Chitosan/TPP	Herring sperm DNA	Calu-3 cells	MTT assay	The cell viability of DNA-loaded chitosan nanoparticles were >70% up to 2 mg/ml after 48 h of incubation.	Ye et al. (2013)
Chitosan low MW	Chitosan/TPP		Human gingival fibroblasts from retromolar tissue	MTS assay LDH assay	Unloaded NPs (100, 300, and 600 µg/ml) did not induce cytotoxic effect, but rather stimulates cell viability and promotes cell proliferation.	Silva et al. (2013)
Chitosan low MW	Chitosan/TPP Chitosan/TPP/Hyaluronic acid		J774.2 cells L929 cells	MTT assay LIVE/DEAD Fluorimetri c assay	All NPs ≤0.1 mg/ml showed >80% cell viability for both cell lines. The hyaluronic acid loaded NPs showed higher cell viability than unloaded NPs.	Nasti et al. (2009)
Chitosan low MW	Chitosan/TPP		Human gingival fibroblasts from retromolar tissue	LDH assay MTS assay	Unloaded NPs showed no cytotoxicity up to 1 mg/ml, reduced cell viability was seen at 5 mg/ml.	Arancibia et al. (2013)
Chitosan medium MW DDA ∼79%	Chitosan/TPP		Bi-potential human liver cells (BHAL)	MTT assay	NPs showed >90% cell viability at pH 7.4 for concentrations up to 1.0% for 4 h, and >70% for 0.5% for 24 h. At pH 6 the cell viability was >90% for 0.1% for both 4 and 24 h.	Loh et al. (2010)
Chitosan medium MW	Chitosan/TPP		Caco-2 cells	MTT assay	NPs showed >80% cell survival at pH 7.4 for concentrations up to 0.1% for 4, 24, 48, and 72 h. For pH 6.4 > 70% survival for 0.025% up to 24 h, and >80% survival for 0.05% up to 48 and 72 h.	Loh et al. (2012)
Chitosan chloride ∼213 kDa	Chitosan/TPP	Ovalbumin	Caco-2 cells	MTS assay LDH assay	MTS assay: Ovalbumin-loaded NPs showed 62% cell viability at 0.1 mg/ml, while 0.05 mg/ml had a reduction of 15%. LDH assay: No cytotoxicity detected for Ovalbumin loaded NPs.	Cole et al. (2018)
Chitosan medium MW< DDA 75%–85%	Chitosan/TPP	Carboxylated 4,4-difluoro-4-bora-3a,4a-diaza-s-indacene (BODIPY-COOH)	A549 cells BEAS 2B cells	MTT assay	Unloaded NPs were non-cytotoxic for both cell types, for all concentrations (0.5, 1, 2, 5, 10, 25 and, 50 µg/ml).	Bor et al. (2017)
Chitosan 404.7 KDa DDA 76%	Chitosan/TPP	Doxorubicin	HT-1197 cells	MTT assay	Unloaded NPs showed no significant decline in cell viability for concentrations ranging from 0,01 to 10 µg/ml.	Ali et al. (2020)
Chitosan chloride MW 307 kDa DDA 83%	Chitosan/TPP		TR146 cells	MTT assay	NPs showed 80% cell viability, and was less cytotoxic than free chitosan.	Pistone et al. (2017b)
Chitosan Hydrochloride DDA 86%	Chitosan/TPP/mannitol		Calu-3 cells A549 cells	MTT assay	Cell viability >80% for all concentrations of NPs (0.001, 0.01, 0.1, 1, 10 mg/ml).	Grenha et al. (2007)
Chitosan chlorhydrate	Chitosan/TPP	Quetiapine fumarate	Goat nasal mucosa	Histology	No observation of cell necrosis or structural damage on nasal mucosa 1 h after administration of quetiapine-fumarate loaded NPs	Shah et al. (2016)
Chitosan DDA 85%	Chitosan/TPP		ATCC CCL 20.2 cells	Trypan Blue SEM	All NP concentrations (0.25, 0.5, 1.0, 2.0 mg/ml) showed >90% viability. SEM showed abundant microvilli and intact membrane details for ≤1.0 mg/ml, for 2 mg/ml a few small membrane holes, some degree of cell flattening and microvilli loss were observed	de Campos et al. (2004)
Chitosan	Chitosan/TPP	Rotigotine	Goat nasal mucosa	Histology	The Rotigotine-loaded NPs produced no toxicity or structural damage to nasal mucosa after 24 h.	Tzeyung et al. (2019)
Chitosan DDA 95%	Chitosan/Tripolyphosphate Chitosan/Phytic acid Chitosan/Sodium hexametaphosphate	Myricetin	Caco-2 cells	MTS assay	Myricetin-loaded NPs showed >90% cell viability for NPs after 24 h for both concentrations (10 and 20 µg/ml).	Sang et al. (2020)

Chitosan	Chitosan/TPP Chitosan/TPP/eudragit	UCN 01 (potent caspase 3 activator)	Caco-2 cells	MTT assay LIVE/DEAD stain	Unloaded NPs showed higher cell viability compared to eudragit-loaded NPs, with >80% cell viability for all concentrations tested (up to 0.5 µM).	Jain et al. (2015)
Chitosan	Chitosan/TPP	Hepatitis E capsid protein p146	L929 fibroblasts	MTT assay	All concentrations of p146-loaded NPs (0–2 mg ml^−1^) showed >80% viability after 24 h.	Wei et al. (2021)
Chitosan	Chitosan/TPP		Gastric tissue from Wistar rats	Macroscopic Histology	Observations indicated that the gastric toxic effects of cadmium chloride were reduced by NPs at 600 mg/kg BW.	Wardani et al. (2018)

No toxicity or structural damage was detected in any of the *ex vivo* studies (Onnainty et al., 2016; Shah et al., 2016; Wardani et al., 2018; Tzeyung et al., 2019). One of them demonstrated that an active ingredient (hydrochlorothiazid) became less toxic when incorporated into chitosan nanoparticles, compared to the free form (Onnainty et al., 2016), and another study showed that chitosan nanoparticles exhibited a protective effect against free radicals (Wardani et al., 2018). An *in vivo* study in mice demonstrated that the chitosan nanoparticles were well tolerated, as no inflammation or pathological changes were detected (Sonaje et al., 2009).

All 25 articles, except one, showed that chitosan nanoparticles (126–1,000 nm) expressed low cytotoxicity (>80% viability) in concentrations ranging from 0.01 to 10,000 µg/ml when evaluated *in vitro*. In the *in vivo* study, up to 100 mg/kg of chitosan nanoparticles were assessed as safe. The only work that showed a somewhat low cell viability was a study where Calu-6 cells were incubated with dry powder chitosan nanoparticles of 250 and 500 µg/ml for 24 h (Dehghan et al., 2013). The Calu-6 cell line is from anaplastic carcinoma with unknown origin, probably the lung. When comparing this finding with the results from another cancer cell line from lungs (Calu-3), the chitosan nanoparticles showed low cytotoxicity at 4 h, and even lower at 48 h (Ye et al., 2013). Recovery of cell viability was also observed in another study, where the cell viability of Caco-2 cells increased from 30% to >80% after 48 h of incubation (Loh et al., 2012). The potential recovery of the Calu-6 cells is not possible to assess because the cells were not incubated for more than 24 h.

### 4.2 Chitosan Nanoparticles Without Tripolyphosphate as Crosslinker

Five articles evaluated the cytotoxicity of chitosan nanoparticles without TPP as crosslinker, consisting of four *in vitro-* and three *in vivo* studies. See Table 2 for main findings and details from the articles on chitosan nanoparticles without TPP as crosslinker. All *in vitro* studies demonstrated good cell viability and low cytotoxicity (Borges et al., 2006; Zhao et al., 2014; Bento et al., 2019; Mumuni et al., 2020). In one of the studies, the nanoparticle-exposed cells showed higher metabolic activity compared to the control, but no cytotoxicity up to 2 mg/ml was detected (Borges et al., 2006).

**TABLE 2:** Articles on chitosan nanoparticles without TPP as crosslinker, main findings.

Chitosan characteristics	Nanoparticle composition	Active ingredient	Cell line/species	Toxicity assay	Cytotoxicity results	References
Chitosan DDA 95%	Chitosan/Na_2_SO_4_ and Chitosan/Na_2_SO_4_/alginate	Ovalbumin	Spleen cells from female BALB/c mice	MTT assay Trypan blue PI stain	No cytotoxicity was detected for any of the unloaded NPs (0.28 and 0.42 mg/ml), on the contrary increased proliferation was observed	Borges et al. (2006)
Chitosan low MW DDA 95%	Chitosan/Na_2_SO_4_	C48/80 (mast cell activator)	Spleen cells from C57BL/6 mice A549 cell line	MTT assay	Spleen cells: >80% cell viability when incubated with unloaded NPs ≤1.08 mg/ml. A549 cell line: >70% cell viability when incubated with unloaded NPs ≤1.5 mg/ml	Bento et al. (2019)
Chitosan 71.3 kDa DDA 80%	Chitosan/Na_2_SO_4_	Newcastle disease virus F gene DNA (pFNDV)	SPF chickens 293-T cells (chicken embryo kidney cellsCEK cells)	Safety test WST-8 kit	Intranasal administration of pFNDV loaded-NPs considered safe. 84% survival rate of kidney cells, no significant changes in cell morphology	Zhao et al. (2014)
Chitosan 200–300 kDa DDA 85%	Chitosan/mucin	Insulin	Wistar rats	Liver enzymes MTT assay	*In vivo*: No significant change in liver enzymes was seen after 3 days of orally administrated 50 IU/kg unloaded NPs.	Mumuni et al. (2020)
					*In vitro*: 0–500 μg/ml showed >98% cell viability after 24 h.	
Chitosan	Chitosan	Chitosan Thymol (2-isopropyl-5- ethylphenol	Nile tilapia fingerlings	Biochemical Macroscopic Histologic	No significant change in survival rate between experimental groups when fed with unloaded and Thymol-loaded NPs.	Abd El-Naby et al. (2020)

Zhao et al. (2014) performed a randomized controlled trial (RCT) to evaluate the *in vivo* safety of the Newcastle-disease-virus-F-gene-DNA vaccine encapsulated in chitosan nanoparticles. Thirty chickens were observed for 3 weeks, showing no clinical symptoms, nervous signs or histopathological changes. The nanoparticles were therefore considered safe (Zhao et al., 2014). This is in agreement with a second RCT where growth and health performance of the Nile tilapia (fish fingerlings) were investigated after adding chitosan and thymol to a basal fish diet (Abd El-Naby et al., 2020). After 70 days, there were no significant changes in survival rate in any of the groups, compared to the control group.

### 4.3 Chitosan in Combination With Liposomes

Liposomes are small artificial sphere-shaped vesicles consisting of one or more phospholipid bilayers. The phospholipids may be derived from natural compounds such as soya and egg, or tissue from bovines, or they can be synthetic. The properties of the liposomes depend on the lipid components. Thus, qualities such as charge, permeability and stability can be engineered. Liposomes have the ability to encapsulate both hydrophilic and hydrophobic substances due to their unique composition with both hydrophilic and hydrophobic parts (Akbarzadeh et al., 2013). Chitosan can interact spontaneously with negatively charged liposomes due to functional amino groups on the chitosan molecule, and by such coat the liposomes (Pistone et al., 2017a).

Five papers concerning the cytotoxicity of liposomes in combination with chitosan were identified; three *in vitro* studies (Adamczak et al., 2016; Klemetsrud et al., 2018; Khan et al., 2019) and two with both *in vivo* and *in vitro* studies (Diebold et al., 2007; Chen et al., 2013a). Cytotoxicity studies regarding nanoparticles with liposomes and chitosan are displayed in Table 3. All the studies used different cell lines and test animals. Four of the articles concluded with low toxicity, high degree of biocompatibility and good tolerance (Diebold et al., 2007; Chen et al., 2013a; Adamczak et al., 2016; Khan et al., 2019). On the contrary, one of the papers demonstrated 10% cell viability after incubation with chitosan coated liposomes (chitosan conc. 0.5%) (Klemetsrud et al., 2018). Interestingly, in another paper, the same nanoparticles but with lower concentration of chitosan (0.1%) showed no reduction in cell viability using both confluent and diluted cell samples in three different cell viability tests (Adamczak et al., 2016). In both papers, the coating of the liposomes was achieved by adding the negatively charged liposomes dropwise into the positively charged chitosan solution, inducing spontaneous formation of chitosan-coated liposomes. Due to up-concentration of the samples in one of the studies the chitosan concentration ended up much higher than in the other. The cell viability results may therefore reflect the chitosan concentration and the amount of potential free chitosan instead of the toxicity of the chitosan coated liposomes.

**TABLE 3:** Articles on chitosan nanoparticles in combination with liposomes, main findings.

Chitosan characteristics	Nanoparticle composition	Active ingredient	Cell line/species	Toxicity assay	Cytotoxicity results	References
Chitosan low MW	Chitosan/lipid	Cisplatin	A2780 cells	Cell Titer Blue assay	Unloaded NPs showed approximately 100% cell viability up to 6.2 µg/ml.	Khan et al. (2019)
Chitosan hydrochloride	Chitosan/SoyPC/EggPG		TR146 cell line	MTS/PMS assay	NPs reduced cell viability of proliferating cells to approximately 10% viability, the cell viability of the stratified cells was around 40%.	Klemetsrud et al. (2018)
Chitosan MW 3.1 × 10^5^ DDA 83%	Chitosan/SoyaPC/EggPG		HT29-MTX cell-line	MTT assay Permeation of paracellular marker	NPs showed high degree of biocompatibility and low toxicity in both confluent monolayer and cells in exponential growth.	Adamczak et al. (2016)
Chitosan 80kD DDA 80%	Chitosan/DOPG/DOPE	Anti-caries DNA vaccine (pGJA-P/VAX)	RAW 264.7 cells Female Balb/c mice	MTT assay Fluorescence imaging	DNA-loaded NPs showed >70% cell viability for concentrations up to 60 µg/ml.	Chen et al. (2013a)
Chitosan hydrochloride	Chitosan/TPP Chitosan/TPP/DSPC/DPPS/CHOL Chitosan/TPP/DSPC/CHOL Chitosan/TPP/DPPS/CHOL		IOBA-NHC cells Female albino New Zealand Rabbit eyeball and lid tissues	XXT assay Macroscopic Histology Cytology	Chitosan NPs showed cell viability >70% for all concentrations (0.25, 0.5, and 1 mg/ml) and incubation times (15, 30 and 60 min), except for 1 mg/ml at 15 min (recovery after 15 min).	Diebold et al. (2007)
					All liposome-chitosan-NPs showed higher cell viability than chitosan NPs. Chitosan NPs and liposomes-chitosan NPs both showed good tolerance *in vivo*.	

**FIGURE 3:**
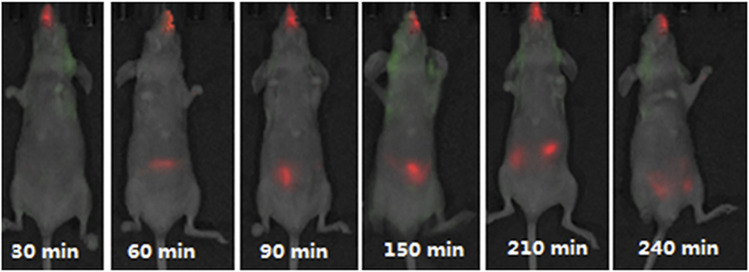
Fluorescence detected in mice after intranasal administration of Cy5.5-marked anionic liposome/chitosan/DNA nanoparticles at different time intervals. Figure adopted from Chen et al. (2013) (https://creativecommons.org/licenses/by/4.0/).

In one of the *in vivo* studies, the passage of fluorescently labelled chitosan/DNA liposomes were traced at different time intervals after intranasal administration in mice (Figure 3). The experiment disclosed nanoparticle clearance via the digestive tract, and no distribution to other organs except the lung was detected (Chen et al., 2013a).

### 4.4 Nanoparticles Coated With Chitosan

Four *in vitro* and one *in vivo* study on the cytotoxicity of nanoparticles coated with chitosan were found. The main results from each study are listed in Table 4. Three of the *in vitro* studies investigated poly (lactic-co-glycolid acid) (PLGA) nanoparticles coated with chitosan. Different cell lines and test animals were used, and the results showed low cytotoxicity and non-irritant properties (Guo et al., 2013; Pandit et al., 2017; Lima et al., 2018). Two of the studies (Guo et al., 2013; Lima et al., 2018) had somewhat conflicting results regarding the non-cytotoxic concentrations (30 vs. 500 µg/ml), but considering that different cell lines were used and that the nanoparticles in one of the studies were loaded with ferulic acid while the others were not, the observed differences should not be overemphasized.

**TABLE 4:** Articles on nanoparticles coated with chitosan, main findings.

Chitosan characteristics	Nanoparticle composition	Active ingredient	Cell line/species	Toxicity assay	Cytotoxicity results	References
Chitosan medium MW DDA 75%–85%	Chitosan/poly (lactic-co-glycolic acid)	Ferulic acid	B16-F10 and HeLa cells	MTT assay	Ferulic acid-loaded NPs showed cell viability 70%–80% for B16-F10 when the concentration was ≤30 µg/ml, and >70% for HeLa cells for up to 60 µg/ml.	Lima et al. (2018)
Chitosan 120 kDa DDA >80%	Chitosan/poly (lactic-co-glycolic acid)	Bevacizumab	Briefly fertilized hen’s eggs	HET-CAM	Unloaded NPs (0.5 ml) were found to be non-irritant as well tolerated for ophthalmic use.	Pandit et al. (2017)
Chitosan DDA 75%–85%	Chitosan/poly (lactic-co-glycolic acid)	7-ethyl-10-hydroxycamptothecin	Caco-2 cells	WST-1 assay LDH-release	Unloaded NPs showed 100% cell viability for concentrations up to 500 µg/ml. A transient effect on the membrane integrity was observed, in a concentration-dependent fashion, but did not have an influence on cell viability.	Guo et al. (2013)
Chitosan	Chitosan/membrane vesicles	*P. salmonis* (ATCC VR 1361) membrane vesicles	Adult zebrafish wild type strain AB	Dose-response experiment Histology	No acute toxic effects were detected in the dose-response experiment, but a reduction in activity levels were observed in fish injected with the highest dose of cMVs (40 µg)	Tandberg et al. (2018)
Chitosan	Chitosan modified mPEG_2000_-b-PCL_4000_-COOH	Tolbutamide	293T cells	MTT assay	Unloaded NPs showed >95% cell viability up to 0.25 mg/ml for 24 h	Shi et al. (2018)

In the *in vivo* study, no mortality or pathological abnormalities were observed in adult zebrafish after injection with bacterial membrane vesicles coated with chitosan (cMVs) (Tandberg et al., 2018).

### 4.5 Derivatives of Chitosan

Several chitosan derivatives have been designed to meet desired requirements and to alter the properties of chitosan. Better solubility and mucoadhesion are the most common requirements. The main drawback of chitosan use has been the low solubility at pH > 6, as this limits its use as a nanocarrier in applications that involve higher pH. Mucoadhesion is also a desirable feature for a nanocarrier for local drug delivery, as it increases the residence time of drugs at the site of action, minimizes the degradation of drugs in various sites and gives the opportunity for a sustained drug release (Ways T. et al., 2018).

#### 4.5.1 Carboxymethyl Chitosan

**FIGURE 4:**
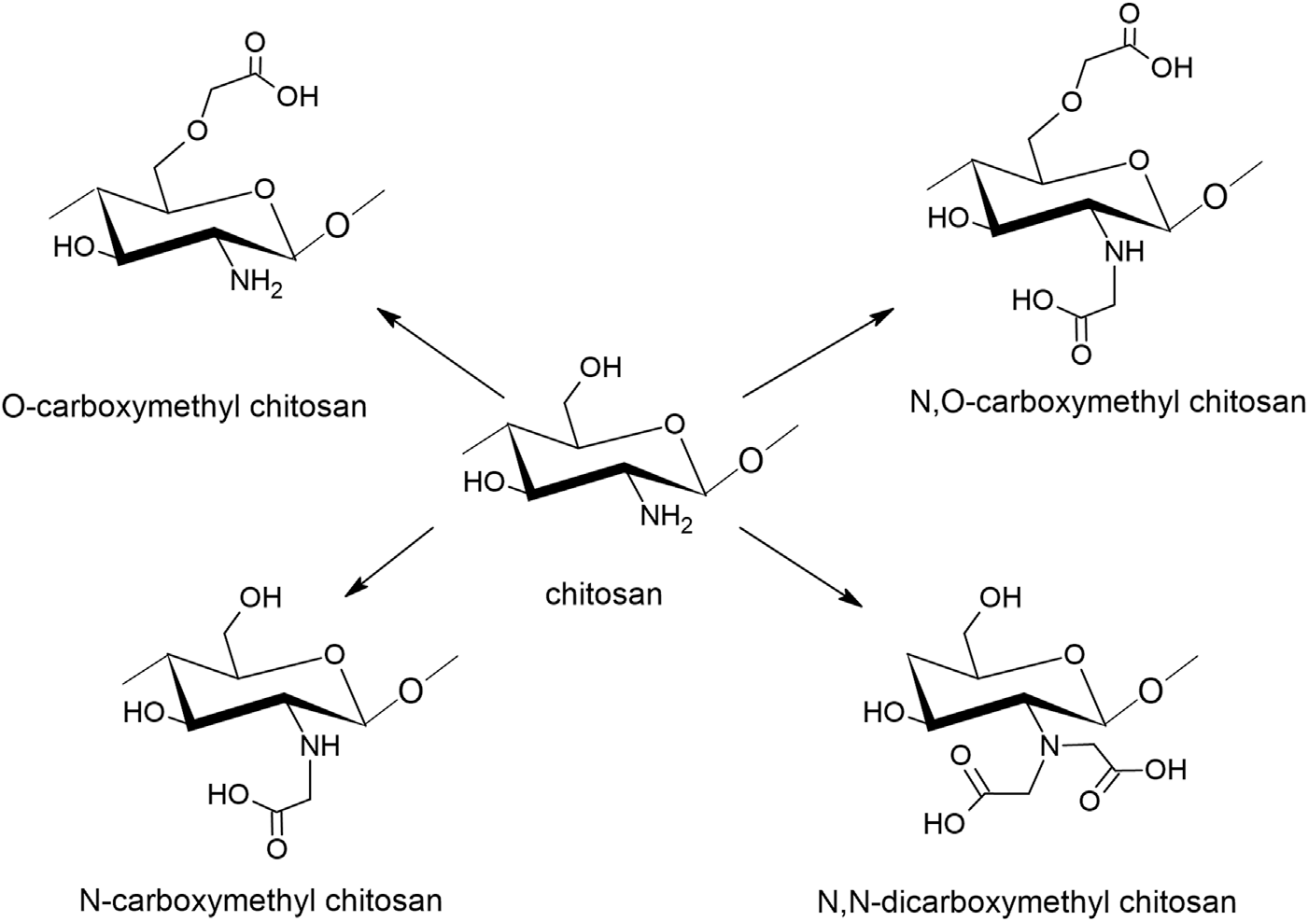
Schematic representation of carboxymethylated chitosan and its derivatives.

To increase its water solubility, chitosan can be chemically modified into carboxymethyl chitosan (CMC) by incorporating negatively charged carboxyl groups to C-6 hydroxyl groups or the NH_2_ group of the glucosamine units, as seen in Figure 4. CMC derivatives are regarded as polyampholytic since they contain both cationic and anionic groups (Chen et al., 2013b). The interest in CMC is rapidly increasing, especially in the biomedical and pharmaceutical field due to its antimicrobial and antioxidant properties. Also in cosmetics, CMC is highly interesting because of the moisturizing and protective effects (Shariatinia, 2018).

Six articles with five *in vitro* and two *in vivo* studies were identified. The main features of the cytotoxicity studies carried out on nanoparticles containing CMC is seen in Table 5. Cytotoxicity of the same nanoparticles were investigated in three of the six papers, using different cell lines and experimental animals (Chakraborty et al., 2010; Chakraborty et al., 2011; Chakraborty et al., 2012). The other three articles used different cell lines and investigated nanoparticles with chitosan of various molecular weights (Liu et al., 2012; Liu et al., 2013; Li et al., 2021). All nanoparticles showed more than 80% cell viability for all tested concentrations, and results from the two *in vivo* studies showed no tissue damage or acute toxicity for any of the tested concentrations (up to 1,000 mg/kg).

**TABLE 5:** Articles on nanoparticles with carboxymethyl chitosan, main findings.

Chitosan characteristics	Nanoparticle composition	Active ingredient	Cell line/species	Toxicity assay	Cytotoxicity results	References
Chitosan 50 kDa, DDA 93.15%, 170 kDa, DDA 92.56%, 820 kDa, DDA 90.14%	Oleoyl-carboxymethyl-chitosan	Fluorescein	Caco-2 cells	MTT assay	Unloaded NPs in all tested concentrations (20, 50, 100, 200, 500, 1,000 µg/ml) showed no cytotoxicity.	Liu et al. (2012)
Chitosan 50 kDa DDA 93.15%	Oleoyl-carboxymethy-chitosan and Oleoyl-carboxymethy-chitosan/hyaluronic acid	Plasmid DNA	Caco-2 cells	MTT assay	Both DNA-loaded NPs showed >90% cell viability for concentrations up to 200 µg/ml.	Liu et al. (2013)
Chitosan medium MW	Carboxymethyl chitosan-2, 2’ ethylenedioxy bis-ethylamine-folate	Vancomycin	NIH 3T3 cells	MTT assay	Unloaded NPs showed no cytotoxicity for concentrations up to 25 µg/ml.	Chakraborty et al. (2010)
Chitosan 53.0 kDa DDA 80%–85%	SiO_2_-Carboxymethyl chitosan-N-2-Hydroxypropyl trimethyl ammonium chloride	Newcastle disease virus	DF-1 cells	CCK-8 assay	Unloaded NPs had an overall survival rate >80% for concentrations up to 1,000 µg/ml.	Li et al. (2021)
Chitosan medium MW	Carboxymethyl chitosan-2, 2’ ethylenedioxy bis-ethylamine-folate		HeLa cells Swiss male mice	MTT assay Acute toxicity	*In vitro*: NPs showed no cytotoxicity for concentrations up to 25 µg/ml.	Chakraborty et al. (2012)
					*In vivo*: The NPs did not cause any mortality up to 1,000 mg/kg and were considered safe.	
Chitosan medium MW	Carboxymethyl chitosan-2, 2’ ethylenedioxy bis-ethylamine-folate		Swiss male mice	Histology Biochemical	Treatment with NPs 1 mg/kg bw/day for 7 days did not cause any kind of tissue damage, alteration of oxidant-antioxidant status or DNA damage of the experimental group.	Chakraborty et al. (2011)

#### 4.5.2 Quaternized Chitosan

Quaternized chitosan is another large group of chitosan derivatives. Both the hydrophilic and mucoadhesive properties of chitosan are improved by quaternization of the primary amino groups. Quaternization of chitosan conserves its positive charge at neutral pH, thus increasing solubility significantly in a much broader pH and concentration range, compared to unmodified chitosan (Kotzé et al., 1999; Thanou et al., 2001). The simplest form of quaternized chitosan is N,N,N-trimethyl chitosan (TMC).

Seven papers that investigated different quaternized chitosan nanoparticles and their cytotoxicity were identified, four with *in vivo* studies (Liu et al., 2015; Jin et al., 2017; Zhao et al., 2017; Yan et al., 2020), two with both *ex vivo* and *in vitro* studies (Amidi et al., 2006; Yin et al., 2009) and one *in vitro* study using three different cell lines (Facchinatto et al., 2021). An overview of cytotoxicity studies concerning nanoparticles with quaternized chitosan is seen in Table 6.

**TABLE 6:** Articles on nanoparticles with quaternized chitosan, main findings.

Chitosan characteristics	Nanoparticle composition	Active ingredient	Cell line/species	Toxicity assay	Cytotoxicity results	References
Chitosan 177 kDa DDA 93%	N-trimethyl chitosan/TPP	Ovalbumin	Calu-3 cells chicken embryo trachea	MTS assay ciliary beat frequency	Unloaded NPs (40 mg/ml) showed >90% cell viability when incubated with Calu-3 cells. Cilio-inhibiting effect: 40% and 80% of the initial value, were seen for unloaded NPs 40 and 8 mg/ml, respectively	Amidi et al. (2006)
Chitosan 30, 200, 500 kDa DDA 85%	Trimethyl chitosan-cysteine	Insulin	Caco-2 cells ileal loop from rats	MTT assay LDH assay	Unloaded NPs (1 mg/ml) demonstrated absence of toxicity for both MTT-assay for Caco-2 cells, and LDH assay on intestinal content from ileal loop.	Yin et al. (2009)
Chitosan 87 kDa DDA 5%	N-(2-hydroxy)-propyl-3-trimethylammonium, O-palmitoyl chitosan (DPCat)	Clotrimazole	HEC-1A endometrial cells CaSki cervical cells HeLa cervical cells	Resazurin assay	Loaded NPs showed >80% cell viability up to 100 µg ml^−1^ in all cell lines, and reduced cytotoxicity compared to free Clotrimazole.	Facchinatto et al. (2021)
Chitosan DDA >95%	N,N,N-trimethyl chitosan chloride (TMC) Alginate-coated TMC (SA-TMC)	Low molecular weight heparin	Male Kunming mice Male SpragueDawley Raw 264.7 macrophages CT26 cells	Histology MTT assay	*In vitro*: Both TMC and SA-TMC unloaded NPs showed >80% viability after 48 h for both cell lines for all concentrations (0–800 μg/ml).	Yan et al. (2020)
					*In vitro*: Loaded TMC and SA-TMC NPs showed no obvious signs of toxicity in main organs after 15 days of 0.1 ml/mg oral administration. In drug-induced colitis, both NPs reversed the effect	
N-2-hydroxypropyl dimethylethyl ammonium chloride chitosan (N-2-HFCC)	N-2-hydroxypropyl dimethylethyl ammonium chloride chitosan/N,O-carboxymethyl chitosan	Newcastle disease virus	Chicken embryonic fibroblast (CEF), 4-week-old SPF chickens	CCK-8 assay Survival rate	*In vitro*: Loaded NPs showed 90% survival rate of CEF cells, with no significant changes in cell morphology.	Jin et al. (2017)
					*In vivo*: The loaded NPs caused little cytotoxicity and had a higher level of biological safety. No difference compared to control groups and no pathological changes were observed.	
N-2-hydroxypropyl trimethyl ammonium chloride chitosan and N,O-carboxymethyl chitosan	N-2-hydroxypropyl trimethyl ammonium chloride chitosan, N,O-carboxymethyl chitosan	Newcastle disease virus and infectious bronchitis virus	Chicken embryo fibroblasts (CEF), 14-days-old chickens	Safety test CCK-8 kit	*In vitro*: Loaded NPs showed a survival rate >90% and no significant changes in cell morphology.	Zhao et al. (2017)
					*In vivo*: The safety test showed that loaded NPs had little cytotoxicity and high safety level, with no difference from the control groups.	
Chitosan low MW	N-trimethylaminoethylmethacrylate chitosan/TPP	Ovalbumin	Sprague-Dawley rats, Blood from New Zealand white rabbit	Histology Inflammatory parameters in rats Percentage of hemolysis in rabbits	Loaded NPs (5 and 25 mg/ml) showed no obvious toxicity to nasal mucosa after administration, no induced oxidative stress or inflammatory reaction. Loaded NPs (0.125–2 mg/ml) induced ≤1% hemolysis, indicating that the loaded NPs will not affect the integrity and functionality of erythrocytes in the blood circulation.	Liu et al. (2015)

The three *in vitro* and *ex vivo* studies showed no cytotoxicity in the specific cell lines or after injection of nanoparticles to the ileal loop of rats. One of the studies measured the reversibility of the ciliary beat frequency in chicken embryo trachea after incubation with TMC. A cilio-inhibiting (25%–75%) effect was seen for the highest concentration (40 mg/ml), but after a concentration adjustment to meet the natural environment (8 mg/ml), the results turned to cilio-friendly (>70%) (Amidi et al., 2006). For all three studies, the nanoparticles showed less cytotoxicity than free TMC.

The four *in vivo* studies showed no obvious toxicity, no pathological changes and no difference in hematological or biochemical parameters from the control group, indicating high level of safety when nanoparticles were administrated intranasally, orally or intramuscularly to mice, rats and chickens (Liu et al., 2015; Jin et al., 2017; Zhao et al., 2017; Yan et al., 2020). In one of the studies, TMC nanoparticles loaded with low molecular weight heparin (LMWH) reversed a drug-induced colitis in mice when the mice were treated orally for 15 days, while mice treated with free LMWH showed no signs of recovery (Yan et al., 2020).

#### 4.5.3 Thiolated Chitosan

Thiolated chitosan is synthesized by covalently coupling sulfhydryl bearing agents such as cysteine, thioglycolic acid or glutathione onto the backbone of chitosan. Thiolated chitosan improves the mucoadhesion properties by forming disulfide units both with glycoproteins of the mucus substrate and the polymer chains (Chen et al., 2013b). The improved mucoadhesive properties make thiolated chitosan attractive for oral delivery of macromolecules. Improved mucoadhesive properties, in combination with permeation properties, enhance the bioavailability of drugs by prolonged residence time and controlled release of the drug (Sakloetsakun et al., 2010; Millotti et al., 2011). As seen in Table 7, the majority of cytotoxicity studies conducted on thiolated chitosan nanoparticles are transmucosal studies with Caco-2 cells.

**TABLE 7:** Articles on nanoparticles with thiolated chitosan, main findings.

Chitosan characteristics	Nanoparticle composition	Active ingredient	Cell line/species	Toxicity assay	Cytotoxicity results	References
Chitosan low MW DDA 75%–85% A549 cells	Aminated chitosan and aminated plus thiolated chitosan	Albumin		MTT assay	Unloaded NPs showed cell viability >80% for concentrations up to 1 mg ml^−1^ in Calu-3 cells, and up to 0.1 mg ml^−1^ for A549 cells. NPs showed higher cell viability than free polymer.	Sinani et al. (2019)
Chitosan low MW DDA 94%	Chitosan- g -Poly (Methyl Methacrylate), thiolated- and unthiolated, crosslinked (TPP) and non-crosslinked (no TPP)		Caco-2 cells HT29-MTX cells (including co-culture model)	MTT assay	Non-crosslinked unthiolated NPs: >80% cell viability of both cell lines, for both concentrations (0.05 and 0.1% w/v) and both times (4 and 24 h), for co-culture model >90%.	Noi et al. (2018)
					Non-crosslinked thiolated NPs: Very variable results, large variance, >80% cell viability for 0.05% w/v in Caco-2 cells, reduced cell viability for 0.1% w/v. For HT29-MTX cells >70% cell viability for 0.05 and 0.1% w/v after 4 h, reduced cell viability after 24 h	
Chitosan water soluble 20 kDa DDA 92%	Poly (isobutylcyanoacrylate) (PIBCA)	Chitosan, chitosan-4- thiol-butylamidine of different ratios	HeLa cells Caco-2/TC7 cells	Trypan blue	HeLa cells: Unloaded and loaded NPs showed low cell viability due to PIBCA core (no chitosan).	Pradines et al. (2015a)
					Caco-2/TC7 cells: Loaded NPs showed >75% cell viability for all ratios up to 50 µg/ml, except 25/75wt% (65%)	
					HT-29/MTX cells: Loaded NPs showed ≥80% cell viability for all ratios up to 50 μg/ml.	
Chitosan 20 kDa DDA 92%	Poly (isobutylcyanoacrylate) (PIBCA) in Pluronic F127 hydrogel	Chitosan, chitosan-4- thiol-butylamidine	Pig vaginal mucosa	Histology	Loaded NPs (75/25 wt%) with concentration 20 mg/ml, did not show any toxicity.	Pradines et al. (2015b)
Chitosan Medium MW DDA 85%	Thiolated chitosan	Centella asiatica	Goat nasal mucosa	Histology MTT assay	*Ex vivo*: No signs of nasal ciliotoxicity.	Haroon et al. (2021)
					*In vitro*: Unloaded NPs of all concentrations (0.0625–625 µ/ml) showed >85% viability.	
Chitosan medium MW DDA 89%	Poly methyl methacrylate, coated with chitosan-glutathione	Paclitaxel	NIH 3T3 cells T47D cells HT29 cells Caco-2 cells	MTT assay	Unloaded NPs of all concentrations (up to 20 µg/ml) showed >80% viability for all cell lines. Thiolation of NPs did not increase the cytotoxicity	Akhlaghi et al. (2010)
Chitosan 60 and 450 kDa	Thiolated chitosan/Sodium alginate	Tizanidine	RPMI 2650 cells	MTT assay	Unloaded NPs up to 40 mg/ml showed no significant toxicity. Thiolation of NPs decreased cytotoxicity.	Patel et al. (2012)
Chitosan 400 kDa DDA 70%–80%	Chitosan-6-mercaptonicotinic acid	Insulin	Caco-2 cells	LDH assay	Unloaded NPs (0–100 µg/ml) showed >90% cell viability. Thiolation of the NPs did not increase cytotoxicity.	Millotti et al. (2011)
Chitosan 400 kDa DDA 70%–85%	Chitosan/TPP/thiobutylamidine	PEG 300, miglyol 840, cremophor EL, caprylic triglyceride	Caco-2 cells	MTT assay LDH assay	Both unloaded and loaded NPs showed >70% viability with both assays.	Sakloetsakun et al. (2010)

Nine articles concerning cytotoxicity of thiolated chitosan nanoparticles were identified, containing nine *in vitro* studies and two *ex vivo* studies, while five of these involved the use of Caco-2 cells (Akhlaghi et al., 2010; Sakloetsakun et al., 2010; Millotti et al., 2011; Pradines et al., 2015a; Noi et al., 2018). All five of these studies showed low cytotoxicity of the thiolated chitosan containing nanoparticles, with the exception of one study that compared non-crosslinked thiolated chitosan nanoparticles to crosslinked thiolated chitosan nanoparticles (Noi et al., 2018). The non-crosslinked as compared to the crosslinked thiolated chitosan nanoparticles expressed very variable cell viability. When the thiolated chitosan nanoparticles were crosslinked, the cell viability increased considerably. The reason for these results may be due to the positively charged surface of the amino group in the non-crosslinked thiolated chitosan that can bind to the negatively charged cell membrane in a cytotoxic manner. In the crosslinked thiolated chitosan, the positively charged surface is neutralized, and the formulation is therefore less cytotoxic. These results are in accordance with previous studies where free chitosan exhibited higher cytotoxicity than crosslinked chitosan, because the charge density of chitosan is reduced by TPP (Pistone et al., 2017b).

Three of the *in vitro* studies also concluded with no, or reduced, cytotoxicity of thiolated chitosan compared to unthiolated chitosan (Akhlaghi et al., 2010; Millotti et al., 2011; Patel et al., 2012). One of the authors explained the results by referring to the higher solubility of thiolated chitosan, and therefore faster removal from the site of application, compared to non-thiolated chitosan (Patel et al., 2012). One of the *ex vivo* studies showed that the herb extract Centella asiatica demonstrated corrosive action comparable to the positive control (isopropyl alcohol) when it was exposed to the nasal mucosa of goats (Haroon et al., 2021). When the same extract was loaded into thiolated chitosan nanoparticles, no erosion or necrosis was detected, and the same results were seen for the unloaded nanoparticles.

In another study, three different cell lines were exposed to chitosan- and thiolated-chitosan coated PIBCA (poly (isobytylcyanoacrylate)) nanoparticles (Pradines et al., 2015a). Both nanoparticles expressed high cytotoxicity towards HeLa cells, but the reason was assumed to be the core nanoparticle (PIBCA) because the same cytotoxicity profile was seen in uncoated PIBCA nanoparticles. The same nanoparticles were investigated *in situ* using pig vaginal mucosa, with no toxicity detected (Pradines et al., 2015b).

### 4.6 Other Derivatives and Complexes With Chitosan

**FIGURE 5:**
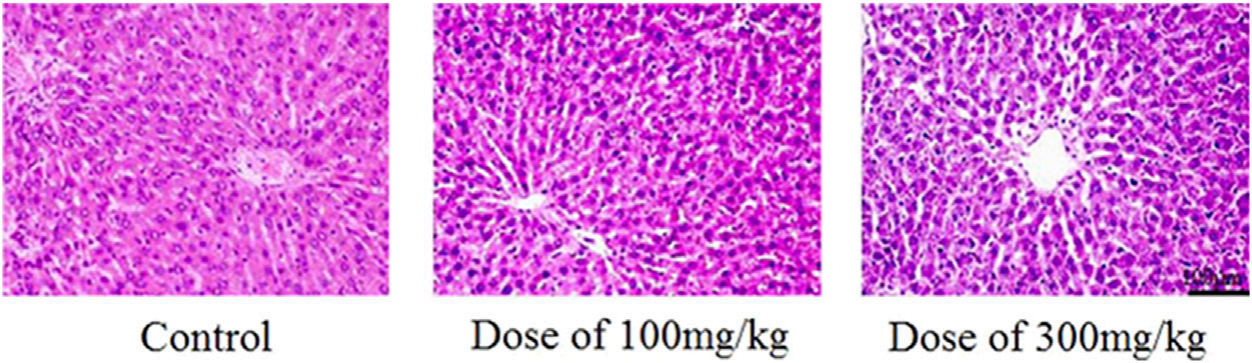
Histological HE-staining of liver from rats after 28 days of oral treatment with and without Alginate/Chitosan/Lovastatin nanoparticles, in two different concentrations (100 and 300 mg/kg). Figure adopted from Thai et al. (2020) (https://creativecommons.org/licenses/by/4.0/).

Eight papers concerning the cytotoxicity of other complexes of chitosan nanoparticles were obtained, five *in vitro* studies (Müller et al., 2000; Cheng et al., 2012; Battogtokh and Ko, 2014; Nguyen et al., 2015; Lim et al., 2018), and three *in vivo* studies (Yan et al., 2019; Mosa et al., 2020; Thai et al., 2020). The complexes in this section are nanoparticles made of chitosan and an active ingredient such as a contrast agent or curcumin, and solid lipid nanoparticles (SLNs) which are hydrophobic nanoparticles based on solid lipid components (Müller et al., 2000). One of the papers investigate the chitosan derivative glycol chitosan and one investigates chitosan nanoparticles with unknown specifications. An overview of the papers on cytotoxicity of nanoparticles of other derivatives and complexes of chitosan, with main findings, is seen in Table 8. The five *in vitro* studies used different cell lines, but they all expressed high cell viability when incubated with the chitosan nanoparticles. The three *in vivo* studies also indicated low toxicity to rats and mice, with no histological changes compared to the negative control, as seen in Figure 5 (Thai et al., 2020). No alterations in hematological or biochemical parameters compared to the control were detected in any of the *in vivo* studies (Yan et al., 2019; Mosa et al., 2020; Thai et al., 2020). The medial lethal dose (LD_50_) of lovastatin loaded nanoparticles was greater than 5,000 mg/kg when administrated orally to mice, and therefore considered nontoxic (Thai et al., 2020).

**TABLE 8:** Articles on other derivatives and complexes with chitosan nanoparticles, main findings.

Chitosan characteristics	Nanoparticle composition	Active ingredient	Cell line/species	Toxicity assay	Cytotoxicity results	References
Chitosan 3–5 kDa DDA 75%	Chitosan/ceramide	Paclitaxel	B16F10 cells MCF-7 cells	MTT assay	Unloaded NPs showed no cytotoxicity in either cell line for concentrations up 100 µg/ml.	Battogtokh and Ko (2014)
Chitosan 50–190 kDa DDA 75–85%	Chitosan/curcumin/hypromellose	Curcumin	NCI-N87 cells	MTT assay	Loaded NPs showed 99.7% and 69% cell viability for Curcumin concentrations of 1 and 10 µg/ml, respectively.	Lim et al. (2018)
Chitosan 50–190 kDa	Chitosan/curcumin	Curcumin	A549 cells	MTT assay	Loaded NPs showed 95 and 85% cell survival for the two Curcumin concentrations of 0.425 and 0.85 mg/ml, respectively.	Nguyen et al. (2015)
Chitosan 80 kDa DDA 95%	Chitosan/Gadolinium	Gadopentetic acid	HeLa cells Male Sprague-Dawley rats	MTT assay	Loaded NPs showed >80% cell survival when Gadolinium concentration was ≤125 µg/ml, for up to 72 h. Loaded NPs below 14.2 mg/ml, administrated rectally and washed out with water after 120 min, were considered safe.	Cheng et al. (2012)
Chitosan 50–60 kDa	Hydroxypropyl trimethyl ammonium/Soybean Lecithin/Glyceryl monostearate	Docetaxel	Caco-2 cells, GI mucosa of Sprague-Dawley rats	MTT assay Histology	Blank SLNs, CS-SLNs, HACC-SLNs all showed >80% at concentration range of 0–2,000 µg/ml. HACC-DTC-SLNs had no toxicity on GI mucosa.	Shi et al. (2017)
Glycol chitosan 168 kDa	5β-cholanic acid-modified glycol chitosan	Fluorescein isothiocyanate-labeled dextrans insulin	Male wistar rats	LDH CII assay BCA Protein Assay	Unloaded NPs of 20 mg/ml did not result in any membrane damage to the jejunum 4 h after jejunal administration.	Yan et al. (2019)
Chitosan powder DDA 75%–85%	Chitosan/alginate	Lovastatin	Thirty adult Swiss mice	Acute- and subchronic toxicity	After 28 days of loaded NP oral injections (0, 100 and 300 mg/kg) no significant differences were seen in hematological- or biochemical parameters and no abnormal signs or mortality were observed. LD_50_ was greater than 5,000 mg/kg and considered practically nontoxic.	Thai et al. (2020)
Chitosan 310–375 kDa DDA 75%	Chitosan	Hydroxyapatite NPs	Eighty male wistar rats	Biochemical parameters, gene expression of oxidant- and antioxidant parameters histology	The test animals were orally treated with 280 mg/kg bw chitosan NPs for 45 days. The chitosan NP treated group showed overall the same or less toxic results compared to the negative control. No histological alterations in the rat small intestine were detected. When chitosan NPs were administrated in combination with hydroxyapatite NPs, the toxic effect from hydroxyapatite NPs was reduced significantly.	Mosa et al. (2020)

In addition to expressing low toxicity in several *in vivo* studies, chitosan nanoparticles (280 mg/kg bw) showed an anti-inflammatory activity by significantly reducing the gastric toxic effect induced by hydroxyapatite nanoparticles in rats (Mosa et al., 2020).

## 5 Discussion

In this overview, 55 papers with *in vitro* studies were identified involving nanoparticles that were exposed to more than 30 different cell lines. Only two studies showed somewhat reduced cell viability after incubation with chitosan nanoparticles (Dehghan et al., 2013; Klemetsrud et al., 2018). Several of the papers demonstrated that chitosan in nanoparticle form was less cytotoxic than chitosan in free form (Amidi et al., 2006; Yin et al., 2009; Pistone et al., 2017b; Facchinatto et al., 2021). The active ingredient (clotrimazole and hydrochlorothiazide) also showed less cytotoxicity when incorporated in chitosan nanoparticles (Onnainty et al., 2016; Facchinatto et al., 2021). Reduced toxicity of the active ingredient (Centella asiatica) was also seen after incorporation into chitosan nanoparticles *ex vivo* (Haroon et al., 2021).

Regarding the *in vivo* studies, all 17 studies showed low toxicity of chitosan nanoparticles independent of administration method, even in high doses (5,000 mg/kg bw). In one of the studies, chitosan nanoparticles even significantly reduced several of the toxic parameters induced by hydroxyapatite NPs (Mosa et al., 2020), and in another study the chitosan nanoparticles exhibited a protective effect against free radicals (Wardani et al., 2018).

The available data regarding the cytotoxicity of chitosan nanoparticles are challenging to compare and summarize due to the vast variation of several factors, such as chitosan properties (molecular weight and deacetylation degree), chitosan derivatives, nanoparticle composition, cell lines, experimental animals and cytotoxicity assays. Several of the collected papers lack details on chitosan properties, such as molecular weight and deacetylation degree, which makes it difficult to draw clear conclusions when it comes to chitosan properties and cytotoxicity.

The pH seems to be an important parameter to consider when evaluating the cytotoxicity, because of its ability to influence particle size and zeta potential. This was demonstrated by Loh et al. (2012) where the viability of Caco-2 cells dropped from 80% to 20% for the same nanoparticles in pH 7.4 and 6.0, respectively. As an example, the pH in the gastrointestinal tract varies from 1 to 8. Therefore, it may be necessary to evaluate the cytotoxicity of nanoparticles in a wide range of pH dependent on the desired exposure route (Jana and Jana, 2020).

Considering the majority of *in vitro* studies, their shortcomings, such as lack of biologic complexity, should be considered and the cytotoxicity results interpreted thereafter. Additionally, the various cell lines may demonstrate different sensitivity towards the same chitosan nanoparticles, as observed in Loh et al. (2010), Loh et al. (2012) where the same nanoparticles showed low viability in Caco-2 cells but good viability in BHAL cells. This was also the case with Klemetsrud et al. (2018) and Adamczak et al. (2016) where the same nanoparticles expressed different cell cytotoxicity towards two different cell lines. The nanoparticles expressed low cytotoxicity towards the mucin producing HT29-MTX cell line, compared to the non-mucin producing TR146 cell line. But the results could also be due to different concentrations of chitosan. *In vitro* models containing multiple cell layers and mucous-like features may mimic biological complexity in a more realistic manner, such as Episkin^TM^ (L’Oréal) or 3D cell culture models (Teubl et al., 2013; De Souza, 2018). Either way, choosing a relevant cell line to the area of use should give results that are more applicable to the final use.

In summary, in spite of all the challenges with comparing the results from different tests and methods, the majority of chitosan nanoparticles demonstrated low cytotoxicity regardless of particle composition, derivatives, cytotoxicity assay, cell lines and animals used in both *in vitro* and *in vivo* studies. Furthermore, chitosan-based nanoparticles have been shown to be less cytotoxic compared to free chitosan, which should strengthen the hypothesis that chitosan nanoparticles are safe. In view of the fact that free chitosan is already on the marked, with increasing demand worldwide, chitosan nanoparticles seem to be a safe and upcoming product. Considering the extensive variation of chitosan and nanoparticle composition in this review, thorough cytotoxicity evaluation should still be performed for all new chitosan-containing nanoparticles in medicine.
